# Associations between *CYP4F2* missense variant rs3093105 A>C and Alzheimer’s disease phenotypes

**DOI:** 10.1002/alz.095286

**Published:** 2025-01-09

**Authors:** Shiropa Noor, Daniel K Mori‐Fegan, Yuen Yan Wong, Che‐Yuan Wu, Richard J. Roman, Ameer Y. Taha, Walter Swardfager, Alzheimer’s Disease Neuroimaging Initiative

**Affiliations:** ^1^ Hurvitz Brain Sciences Program, Sunnybrook Research Institute, Toronto, ON Canada; ^2^ University of Toronto, Toronto, ON Canada; ^3^ Sandra Black Centre for Brain Resilience and Recovery, Sunnybrook Research Institute, Toronto, ON Canada; ^4^ University of Mississippi Medical Center, Jackson, MS USA; ^5^ University of California, Davis, Davis, CA USA

## Abstract

**Background:**

20‐hydroxyeicosatetranoic acid (20‐HETE) is a potent vasoconstrictor synthesized by the CYP4F2 enzyme. The *CYP4F2* missense variant rs3093105 A>C (W12G) has been implicated in hypertension and stroke, risk factors for Alzheimer’s disease (AD), and with decreased 20‐HETE activity. To explore the potential role of the CYP4F2/20‐HETE pathway and AD, this study investigated associations between the rs3093105 variant and AD phenotypes.

**Method:**

Participants from the Alzheimer’s Disease Neuroimaging Initiative with amyloid‐beta 42 positive PET status and a diagnosis of AD or mild cognitive impairment were selected. Linear regressions explored the relationship between rs3093105 C‐allele carrier status (AC/CC vs AA) and cognition, assessed as executive function and memory composites and Rey Auditory Verbal Learning Test (RAVLT) scores; and brain volumetric measures at baseline, obtained using FreeSurfer on MRI T1‐weighted images. Longitudinal associations (up to 48 months) were explored via interactions between carrier status and time in mixed effects models. Covariates included baseline age, sex, APOE e4 status, years of education, intracranial volume, and Mini Mental State Exam score.

**Result:**

The rs3093105 variant was present at a minor allele frequency of 19.2% in 315 individuals (mean age 73.3 ± 7.57, 43.2% women). At baseline, C‐allele carriers had higher executive function composite scores (B = 0.315, SE = 0.119, *p* = 0.009, *n* = 315) and RAVLT immediate (sum of trials 1‐5) scores (B = 2.46, SE = 1.24, *p* = 0.048, *n* = 315), and larger whole brain volume (B = 14.19, SE = 6.84, *p* = 0.039, *n* = 310). Over time, carriers had higher memory composite scores (B = 0.116, SE = 0.052, *p* = 0.027, *n* = 183) and RAVLT learning (trial 5 minus trial 1) scores (B = 0.484, SE = 0.207, *p* = 0.021, *n* = 179), and retention in hippocampal volume (B = 4.19, SE = 1.71, *p* = 0.016, *n* = 127).

**Conclusion:**

Altogether, rs3093105 C‐allele carriers show better performance on memory tests and retention of hippocampal volume. These findings suggest a potential role of the CYP4F2/20‐HETE pathway in AD progression and warrants replication studies in other cohorts.

